# Effect of Heat-Input on Microstructure and Toughness of CGHAZ in a High-Nb-Content Microalloyed HSLA Steel

**DOI:** 10.3390/ma15103588

**Published:** 2022-05-18

**Authors:** Hongwei Yu, Kaiming Wu, Baoqi Dong, Jingxi Liu, Zicheng Liu, Daheng Xiao, Xing Jin, Hankun Liu, Minmin Tai

**Affiliations:** 1 The State Key Laboratory of Refractories and Metallurgy, Wuhan University of Science and Technology, Wuhan 430081, China; yuhongwei@baowugroup.com (H.Y.); dongbq2016@163.com (B.D.); 2 School of Naval Architecture and Ocean Engineering, Huazhong University of Science and Technology, Wuhan 430074, China; liu_jing_xi@hust.edu.cn; 3 Department of Manufacturing, Baoshan Iron & Steel Co., Ltd., Shanghai 201999, China; liuzc@baosteel.com; 4 Technology Center, Hunan Valin Xiangtan Steel Co., Ltd., Xiangtan 411101, China; xiaodh123@126.com; 5 Department of Manufacturing, Nanjing Iron & Steel Co., Ltd., Nanjing 210044, China; jinxing@njsteel.com.cn; 6 China Petroleum Group Ocean Engineering (Qingdao) Co., Ltd., Qingdao 266520, China; liuhk.cpoe@cnpc.com.cn; 7 Shandong Marine Group Ltd., Jinan 250102, China; taimm2022@163.com

**Keywords:** CGHAZ, thermal cycle, impact toughness, niobium microalloying

## Abstract

The effect of various heat inputs on the microstructure and impact toughness of the simulated coarse-grained heat-affected zone (CGHAS) of a niobium microalloyed (0.14 wt.%) low-carbon steel was studied. The results showed that higher impact toughness was achieved at a low heat input of 20 kJ/cm, which resulted from the formation of acicular ferrite laths/plates. They sectioned large prior austenite grains into many smaller regions, resulting in smaller crystallographic grains and high-angle grain boundaries. Conversely, when specimens were simulated with larger heat-inputs (100, 200 kJ/cm), the microstructure of the CGHAZ was predominantly composed of granular bainite plus massive MA constituents, thus impairing the impact toughness.

## 1. Introduction

Due to the good balance of strength, toughness and excellent elongation, high-strength low-alloy (HSLA) steels are used to construct bridges, pipelines, pressure vessels and offshore drilling platforms [1,2,3,4]. The production of high toughness steel is an essential requirement due to its utilization in extreme environments such as in the Gulf of Alaska and the North Sea for the search of gas and oil [4,5], but it is still difficult to achieve superior toughness when the strength exceeds 700 MPa at relatively low costs [6].

Nb as microalloying element has gained attention due to its special properties of toughening and strengthening HSLA steels, especially those made by the thermo-mechanical control process (TMCP). The addition of Nb in steel effectively improves the strength, as mentioned in many studies [7,8,9], as the addition of a small amount of Nb forms carbonitride, which refines prior austenite grains. Additionally, Nb not only improves the mechanical properties, but also act as a corrosion resistance element in the atmosphere and sea water [10,11,12,13,14,15].

In spite of the fact that Nb improves the toughness and strength of the base plate, it is also reported to weaken the toughness of the heat-affected zone (HAZ) because of martensite hardening [16,17]. Nb restricts the heterogenous acicular ferrite nucleation on unstable carbonitrides, and helps in coarse upper bainitic microstructure formation at low cooling rates (t_8/5_ ≥ 50 s). Some studies [18,19] indicated that with increasing carbon content, the micro-segregation of Mn and Nb occurs, thus increasing the hardenability of the re-austenitized structure during the second thermal cycle through promoting martensite/retained austenite (MA) constituent formation [20]. Moreover, during the third thermal cycle, it obstructs MA decomposition by means of preventing carbon atom diffusion. The cycle loading under the stress concentration of CGHAZ was also investigated. It was found that the fatigue property of the CGHAZ is low compared with base metal [21]. Recently, Kryzhanivskyy et al. [22] investigated the toughness of an X70 pipeline steel containing 0.10% Nb. It was found that the impact toughness of the investigated pipeline steel was very high, although the impact toughness of the fusion zones was 1.14~1.5 times lower compared with the base metal.

The impact of Nb on HAZ toughness depends significantly on the carbon content and amount of Nb utilized. Low-Nb content, i.e., ≤0.02%, was suggested to improve the toughness of the heat-affected zone in high-strength TMCP steels. When the content of carbon is low (i.e., 0.03–0.04 wt.%), a Nb content above 0.04% is utilized without harmful effects on the toughness of the heat-affected zone, and is usually adopted in TMCP-treated steel to retard and prevent the softening of the heat-affected zone [9]. Zhang et al. investigated the influence of heat input on CGHAZ toughness in a low-Nb-bearing (i.e., 0.026 wt.%) microalloyed HSLA steel [23]. The results showed that the microstructure was composed of granular bainite and lath bainite at a heat input of 100 kJ/cm. The volume percentage of granular bainite increased to a full 100% when the heat input was lowered to 60 kJ/cm. A significant amount of lath martensite formed when the heat input was further reduced to 30 kJ/cm. It was also reported that in steel with a high content of Nb and C, the micro-segregation of Nb and Mn and the carbon segregation in un-transformed austenite near grain boundaries was seen to be responsible for the formation of MA constituent [24,25]. Therefore, the effect of different amounts of Nb on the base metal and HAZ have been extensively investigated [26,27]. However, limited investigation has been performed on the influence of high Nb content on the properties and microstructure of CGHAZ.

The present work aims to study the influence of high Nb content on toughness and microstructure during welding simulation with varying heat inputs. The influence of various heat inputs on microstructural features such as grain boundary misorientation, crystallographic grain size and MA constituent in a high-Nb-content (0.14 wt.%) microalloyed steel has been investigated, with the aim of providing generic guidelines in the design and welding of Nb-microalloyed HSLA steels.

## 2. Experimental Procedures

### 2.1. Material

The steel plate was industrially produced via continuous casting and TMCP, followed by a tempering process. The raw material was taken from the industrially produced plate. It was remelted with the addition of Nb in a vacuum smelting furnace and cast into an ingot. The fully solidified ingot was forged into 15 mm plates in a pilot plant. Chemical compositions of the simulated steel were analyzed using an optical emission spectrometer (Shimadzu, PDA-7000). The obtained results are shown in Table 1. All concentrations are given in wt.%. The equivalent carbon content (Ceq) is 0.3219 and the welding crack susceptibility index (Pcm) is 0.1391.

### 2.2. Simulation of CGHAZ

Approximately 20 kJ/cm is usually used in industrial welding, for example, in submerged arc welding and gas shielding arc welding (CO_2_ + Ar). In electro-gas welding, 200 kJ/cm is more common. Therefore, 20 kJ/cm is selected to simulate submerged arc welding, gas shielding arc welding, etc., whereas 200 kJ/cm is used to simulate electro-gas welding. In addition, 100 kJ/cm is an intermediate level of heat input for other welding techniques. In order to obtain different CGHAZs with heat inputs (E) of 20, 100 and 200 kJ/cm, the simulation of CGHAZs was conducted using Gleeble 3800 (Dynamic System Inc., Poestenkill, NY, USA) for different cooling rates of *t*_8/5_ (i.e., from 800 to 500 °C). The size of the specimens was 11 × 11 × 55 mm^3^.

The relation between heat input *E* and cooling time *t*_8/5_ (from 800 to 500 °C) is given in Equation (1) [28].

(1) $$t_{8/5}=(0.67-5\times 10^{-4}T_{0})E(\frac{1}{500-T_{0}}-\frac{1}{800-T_{0}})$$

where *T*_0_ is the initial temperature (20 °C). The *t*_8/5_ were 10.6 s, 52.8 s and 105.6 s, which corresponded to the different welding heat inputs (20, 100, 200 kJ/cm).

**Figure 1:**
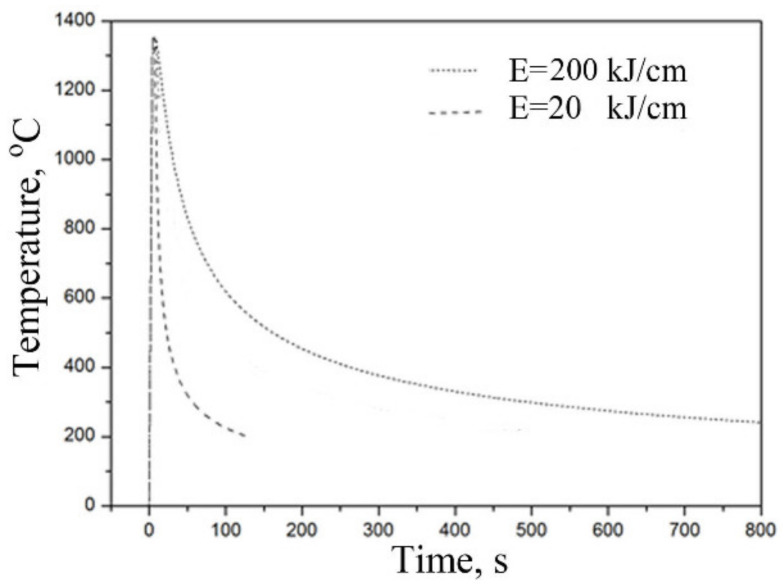
Thermal cycles used in the simulation of CGHAZ.

Four parameters may influence simulation: heating rate from initial temperature to peak temperature (R_h_), peak temperature (T_p_), holding time at peak temperature (t_h_) and cooling time 800–500 °C (*t*_8/5_). The samples were rapidly heated at 300 °C/s to peak temperature (1350 °C), then held for 3 s, and subsequently cooled at various cooling rates. The time of *t*_8/5_ is to cool the specimen from 800 °C to 500 °C. The simulation parameters are listed in Table 2, and the thermal cycles for CGHAZ simulation are presented in Figure 1.

### 2.3. Microstructural Features and Toughness Measurements

Specimens were prepared utilizing standard metallographic methods and etched with a nital solution of 4 vol.%. To reveal the MA constituent, the polished specimens were etched in two steps. First, the specimens were electrolytically etched for 10 s at 3 V in the solution consisting of a mixture of 5 g tetra-acetic acid (EDTA) and 0.5 g NaF in distilled water with a volume of 100 mL. Next, they were electrolytically etched for 60 s at 6 V in a solution consisting of a mixture of 5 g picric acid and 25 g NaOH in 100 mL distilled water.

Semi-automatic electrolytic polishing etching equipment was used to electrolytically polish the pre-polished specimens. The detailed microstructure observations were made by scanning electron microscopy (SEM). The intercept method, according to the ASTME112-96, was utilized for austenite grain-size measurements. More than 30 fields were observed on the polished sample surface by an optical microscope at a magnification of 200. Electron backscatter diffraction (EBSD) was used for analyzing grain boundary misorientation, crystallographic grain size, etc.

The impact toughness (V-notch Charpy) tests (JB-300B) were performed at −20 °C using standard specimens (Standard ASTM E23). The absorbed energy of three tests was measured for each specimen.

## 3. Results

### 3.1. Microstructure

#### 3.1.1. Optical Microstructure and Austenite Coarsening

**Figure 2:**
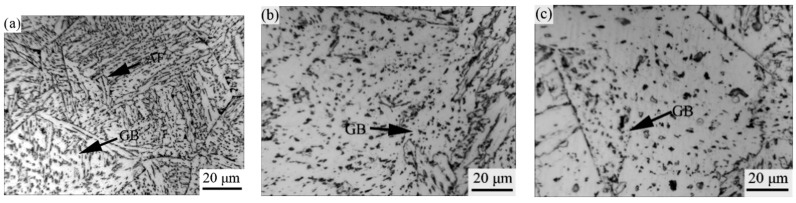
Optical images of the CGHAZ containing 0.14% Nb at varying heat inputs (kJ/cm). (**a**) 20, (**b**) 100, (**c**) 200.

Figure 2 presents optical micrograph images in the CGHAZ of the 0.14 wt.% Nb steel with various heat inputs. It is clear that the microstructure of CGHAZ at a heat input of 20 kJ/cm mainly consisted of bainitic ferrite and acicular ferrite. When the heat input was increased to 100 kJ/cm, the microstructure of the CGHAZ comprised of granular bainite and a high fraction of bainitic ferrite. It is seen that granular bainite has large packets dispersed with island-like MA constituents. As the heat input was further increased up to 200 kJ/cm, the CGHAZ microstructure was mainly coarse granular bainite. This result is in agreement with that obtained with high heat input (60–100 kJ/cm) for a low-Nb-content (0.026%) steel [22]. Additionally, it is clear that with the increase of heat input, the size of the prior austenite grains also increased (Table 3).

#### 3.1.2. MA Constituent

**Figure 3:**
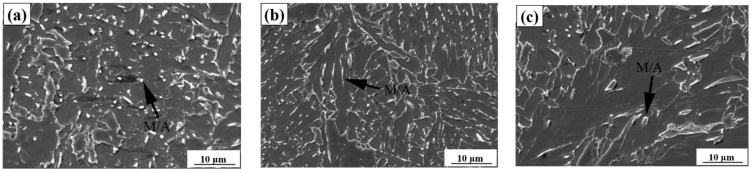
SEM images of the CGHAZ containing 0.14% Nb at varying heat inputs (kJ/cm). (**a**) 20, (**b**) 100, (**c**) 200.

**Figure 4:**
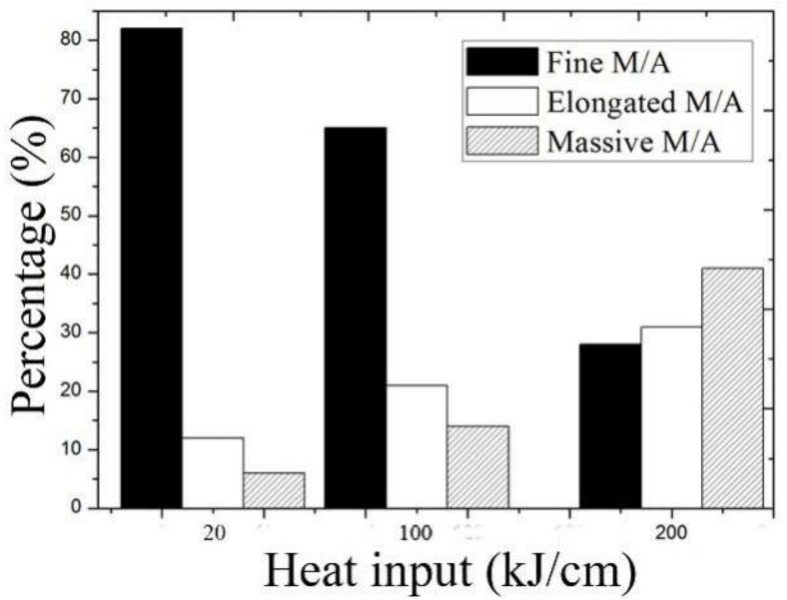
Histogram of MA distribution in the CGHAZ containing 0.14% Nb.

Figure 3 shows scanning electron microscope images in the specimens simulated at various heat inputs. The histogram shows the MA constituent distribution in CGHAZs (Figure 4 and Table 4). It is clear that for the sample simulated with a heat input of 20 kJ/cm, the MA constituent was fine and evenly distributed in the steel matrix. The percentage of fine MA was 82%, whereas massive and elongated MA were 6% and 12%, respectively. As the heat input further increased to 100 kJ/cm, the elongated MA constituent increased up to 21%. As the heat input continued to increase up to 200 kJ/cm, there was a significant increase in massive MA and a decrease in fine MA. The percentage of massive MA was 41%, whereas the fine MA was 28%.

#### 3.1.3. Crystallographic Features

**Figure 5:**
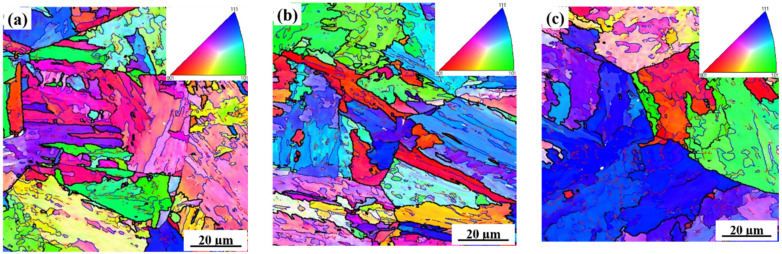
Orientation maps of α phase in normal direction of microstructure in the CGHAZ containing 0.14% Nb at varying heat inputs (kJ/cm). (**a**) 20, (**b**) 100, (**c**) 200.

The bcc-phase orientation maps with varying heat inputs (20, 100, 200 kJ/cm) are presented in Figure 5. Table 3 shows the measured size of the crystallographic grains in the CGHAZ. From Figure 5 and Table 3, it is clear that with an increase in heat input, the crystallographic grain size become larger: the grain size increased from 7.5 µm with a low heat input (20 kJ/cm) to 11.0 µm with a high heat input (200 kJ/cm). Table 5 provides the results of a grain-boundary misorientation angle between neighboring grains. It is clear that the high fraction of high-angle grain boundaries (>10°) was maintained in the specimens simulated with a low-heat input (20 kJ/cm) compared to the specimens simulated with high-heat inputs (100 kJ/cm, 200 kJ/cm).

#### 3.1.4. Impact Toughness

Table 6 presents the V-notch impact absorbed energy values of the specimens simulated with various heat inputs (20, 100, 200 kJ/cm). The average absorbed energy at −20 °C was 166 J at a heat input of 20 kJ/cm, while it dropped to 10 J at a heat input of 200 kJ/cm. It is clear that the average of the CGHAZ impact toughness dropped with increasing heat inputs and the toughness decreased to a very low value at a high-heat input (200 kJ/cm).

## 4. Discussion

### 4.1. Influence of Heat Input on Microstructural Change

It is well known that the microstructure becomes coarse when ordinary carbon steels are welded at a high-heat input. Therefore, the concept of oxide metallurgy and microalloying has been well established in the last decades [5]. Nb is an important microalloying element in steels, and can form carbonitride particles that pin grain boundaries and inhibit grain coarsening [9]. In the present study, Nb microalloying was adopted and high-heat input was considered. The nano-sized carbonitride particles formed in the steel matrix can induce a pinning effect on grain boundaries and retard grain coarsening at a low-heat input (20 kJ/cm). However, when the heat input further increased to 200 kJ/cm, the time for maintaining a high temperature became longer, and the particles became coarse in such a way that the pinning effect on the grain boundaries was reduced. The austenite grain size was greatly increased and the microstructure was coarsened at a high-heat input (200 kJ/cm) compared to a low-heat input (20 kJ/cm) (see Table 3 and Figure 2).

### 4.2. Influence of Heat Input on MA Formation

MA constituent is an important phase transformed in the CGHAZ of HGLA steels. Besides steel composition, the shape and amount of MA constituent mainly depends on the cooling rate, which is strongly correlated with heat input, specifically for heavy plates. Under low-heat input (20 kJ/cm), many fine MA constituents were obtained in the steel matrix. The fine MA constituents can change the cracking direction and retard dislocation movement and cracking propagation [28], and is therefore good for strength and toughness. Thus, when the simulation was carried out with a low-heat input (20 kJ/cm), the impact toughness of the CGHAZ was high (see Table 6). As the heat input increased to 200 kJ/cm, the granular bainite increased and became coarse; thus, massive MA constituent was produced in the granular bainite. Meanwhile, the MA constituent can also become coarse and massive in shape and be present at grain boundaries (see in Figure 3), which is detrimental to toughness [12]. These factors effectively reduce crack propagation energy and deteriorate the toughness of the CGHAZ [29]. Thus, the absorbed impact energy decreased to a significantly low level at a high-heat input (200 kJ/cm) (see Table 6).

### 4.3. Influence of Heat Input on Acicular Ferrite Formation

Numerous factors are reported to influence acicular ferrite formation. Types of inclusions, steel composition, and prior austenite grain size are usually considered to be important factors. In this study, the oxides precipitated during steel-making and Nb-Ti carbonitride particles formed during cooling provided nucleation sites for acicular ferrite. The size of the prior austenite grains is also significant for acicular ferrite formation. An optimum size of 50–110 μm was reported to be beneficial for acicular ferrite formation [30]. In the present study, the size of the austenite grains was about 87 and 99 μm, corresponding with a heat input of 100 and 200 kJ/cm, respectively. Many AF laths/plates were transformed in the CGHAZ (Figure 2a). The larger austenite grains formed during high-heat input welding are sectioned by AF plates or laths formed on inclusions into much smaller regions. The subsequent microstructure formation is restricted in these small regions and, thus, the final microstructure is refined. The improvement in toughness is therefore realized. In the meantime, if the size of the austenite grains is too large, or the number of AF plates or laths is not enough, the refinement of the final microstructure is not very efficient (Figure 2a,c).

Besides the grain refinement caused by acicular ferrite formation, the crystallographic misorientation of the grain boundary also affects toughness [9]. From Table 5, it can be seen that with the decrease of the heat input, the high-angle grain boundary (≥10°) was increased. It can also be seen that the sample simulated at a heat input of 100 kJ/cm had higher toughness than that of 200 kJ/cm. The improvement in toughness is attributed to more high-angle grain boundaries, which can change the cracking direction and obstruct cracking propagation [9].

### 4.4. Influence of Heat Input on Grain Boundary Misorientation

From Table 5, it can be seen that the percentage of high-angle grain boundaries was greater at low-heat input (20 kJ/cm); however, it decreased at high-heat input (100, 200 kJ/cm). At low-heat input, a fine microstructure (Figure 2a) was formed with a higher cooling rate. This means that many high-angle grain boundaries were obtained (Table 5). However, the microstructure was transformed into coarse granular bainite at high-heat input (100, 200 kJ/cm). The granular bainitic microstructure had a similar misorientation with low-angle grain boundaries. Given that a high-angle grain boundary can change cracking direction and efficiently inhibit cracking propagation [9], the toughness at low-heat input (20 kJ/cm) was high, but it dropped significantly at high-heat input (200 kJ/cm).

Besides the high-angle grain boundary, acicular ferrite can also influence impact toughness [9]. From Figure 2a, it is clear that there were some acicular ferrite plates or laths present in the final microstructure. AF plates or laths can divide large austenite grains into smaller regions, thus refining the microstructure and improving toughness.

## 5. Conclusions

(1) The influence of heat inputs on the microstructure and impact toughness of the simulated CGHAZ of a high Nb-content (0.14%), high-strength steel was investigated. When the steel was simulated with a low-heat input of 20 kJ/cm, many acicular ferrite laths/plates formed in the CGHAZ. The lath/plate-like acicular ferrite sectioned the large austenite grains into many small and separate regions, thus resulting in smaller-sized crystallographic grains and high-angle grain boundaries, such that the toughness was high.
(2) When the steel was simulated with higher heat inputs (100, 200 kJ/cm), the microstructure of the CGHAZ was predominantly composed of coarse granular bainite and massive MA constituents. The deleterious toughness in CGHAZ with higher heat inputs was mainly caused by the coarser microstructure of the granular bainite and massive MA constituents.

## Figures and Tables

**Table 1:** The chemical analysis of tested steel samples (wt.%).

C	Mn	Si	Nb	V	Al	Ti	Fe
0.049	1.61	0.22	0.14	0.023	0.026	0.013	Balance

**Table 2:** Welding simulation parameters.

E	R_h_	T_p_	t_h_	t_8/5_
20 kJ/cm	300 °C/s	1350 °C	3 s	10.6 s
100 kJ/cm				52.8 s
200 kJ/cm				105.6 s

**Table 3:** Measurements of austenite grain size in CGHAZ.

E, kJ/cm	Size of Austenite Grains (μm)	Size of Crystallographic Grains (µm)
20	57.3 ± 5.68	7.5 ± 0.25
100	86.7 ± 4.28	10.0 ± 0.42
200	98.9 ± 4.36	11.0 ± 0.23

**Table 4:** Distribution of fine, elongated and massive MA constituents in the CGHAZ containing 0.14% Nb.

E, kJ/cm	Distribution of A Constituents		
	Fine MA	Elongated MA	Massive MA
20	82%	12%	6%
100	65%	21%	14%
200	28%	31%	41%

**Table 5:** Percentage of different grain misorientation angles.

E, kJ/cm	<3° (%)	>5° (%)	>10° (%)	>15° (%)	>30° (%)	>40° (%)
20	50.92	42.71	30.71	26.3	24.03	23.42
100	59.82	32.18	16.21	12.9	11.8	11.6
200	61.67	31.15	12.34	10.71	9.9	9.7

**Table 6:** Measured impact toughness results of the CGHAZ containing 0.14% Nb at −20 °C.

E, kJ/cm	Absorbed Energy for Charpy V-Notch Impact Test, J		
	Max	Mean	Min
20	190	166	144
100	102	82	60
200	11	10	8

## Data Availability

Not applicable.
